# Medical treatment versus “Watch and Wait” in the clinical management of CE3b echinococcal cysts of the liver

**DOI:** 10.1186/1471-2334-14-492

**Published:** 2014-09-09

**Authors:** Francesca Rinaldi, Annalisa De Silvestri, Francesca Tamarozzi, Federico Cattaneo, Raffaella Lissandrin, Enrico Brunetti

**Affiliations:** Department of Clinical-Surgical, Diagnostic and Paediatric Sciences, WHO Collaborating Centre for Clinical Management of Cystic Echinococcosis, University of Pavia, via Brambilla 74, 27100 Pavia, Italy; Department of Infectious Diseases, San Matteo Hospital Foundation, via Taramelli 5, Pavia, 27100 Italy; Biometry Unit, San Matteo Hospital Foundation, P.le Golgi 19, Pavia, 27100 Italy

## Abstract

**Background:**

Available treatments for uncomplicated hepatic cystic echinococcosis (CE) include surgery, medical therapy with albendazole (ABZ), percutaneous interventions and the watch-and-wait (WW) approach. Current guidelines indicate that patients with hepatic CE should be assigned to each option based on cyst stage and size, and patient characteristics. However, treatment indications for transitional CE3b cysts are still uncertain. These cysts are the least responsive to non-surgical treatment and often present as indolent, asymptomatic lesions that may not warrant surgery unless complicated. Evidence supporting indications for treatment of this stage is lacking. In the attempt to fill this gap before the implementation of randomized clinical trials, we compared the clinical behavior of single hepatic CE3b cysts in 60 patients followed at the WHO Collaborating Centre for Cystic Echinococcosis of the University of Pavia.

**Methods:**

We analyzed retrospectively data of 60 patients with hepatic CE3b cysts seen at our clinic over 27 years, who either received ABZ or were monitored with WW. Univariate and multivariate analysis were performed to investigate the effect on outcome (inactivation or relapse) of variables such as age, sex, origin, treatment, cyst size and presence of other echinococcal hepatic cysts using a multiple failure Cox proportional hazard model.

**Results:**

ABZ treatment was positively associated with inactivation (p < 0.001), but this was not permanent, and no association was found between therapeutic approach and relapse (p = 0.091). No difference was found in the rate of complications between groups.

**Conclusions:**

In conclusion, our study shows that ABZ treatment induces temporary inactivation of CE3b cysts, while during WW cysts remain stable over time. As the rate of adverse events during periods of ABZ treatment and WW did not differ significantly in the follow-up period considered in this study (median 43 months, IQR 10.7-141.5), expectant management might represent a valuable option for asymptomatic CE3b cysts when strict indication for surgery is absent and patients comply with regular long-term follow-up.

**Electronic supplementary material:**

The online version of this article (doi:10.1186/1471-2334-14-492) contains supplementary material, which is available to authorized users.

## Background

Cystic Echinococcosis (CE), caused by the zoonotic tapeworm *Echinococcus granulosus*, is a serious health problem in many regions of the world, but is among the most neglected parasitic diseases [1]. The liver is the most frequent location of echinococcal cysts (70% of cases), with a highly variable clinical presentation, ranging from asymptomatic to life-threatening manifestations [2, 3]. The current management of CE is based on quality of evidence III (evidence from opinions of respected authorities, based on clinical experience, descriptive studies, or reports of committees) with B-D recommendation strength (moderate to poor evidence to support recommendation). This situation results from the lack of longitudinal controlled studies, which is partly due to (1) the chronicity of the disease which requires years-long follow-up to evaluate the effectiveness of an intervention, (2) the relatively low number of patients presenting with clinically homogeneous conditions, even in referral centres in endemic countries, such our WHO collaborative centre in Italy, and (3) to the difficulty in comparing results from different studies [4].

**Figure 1:**
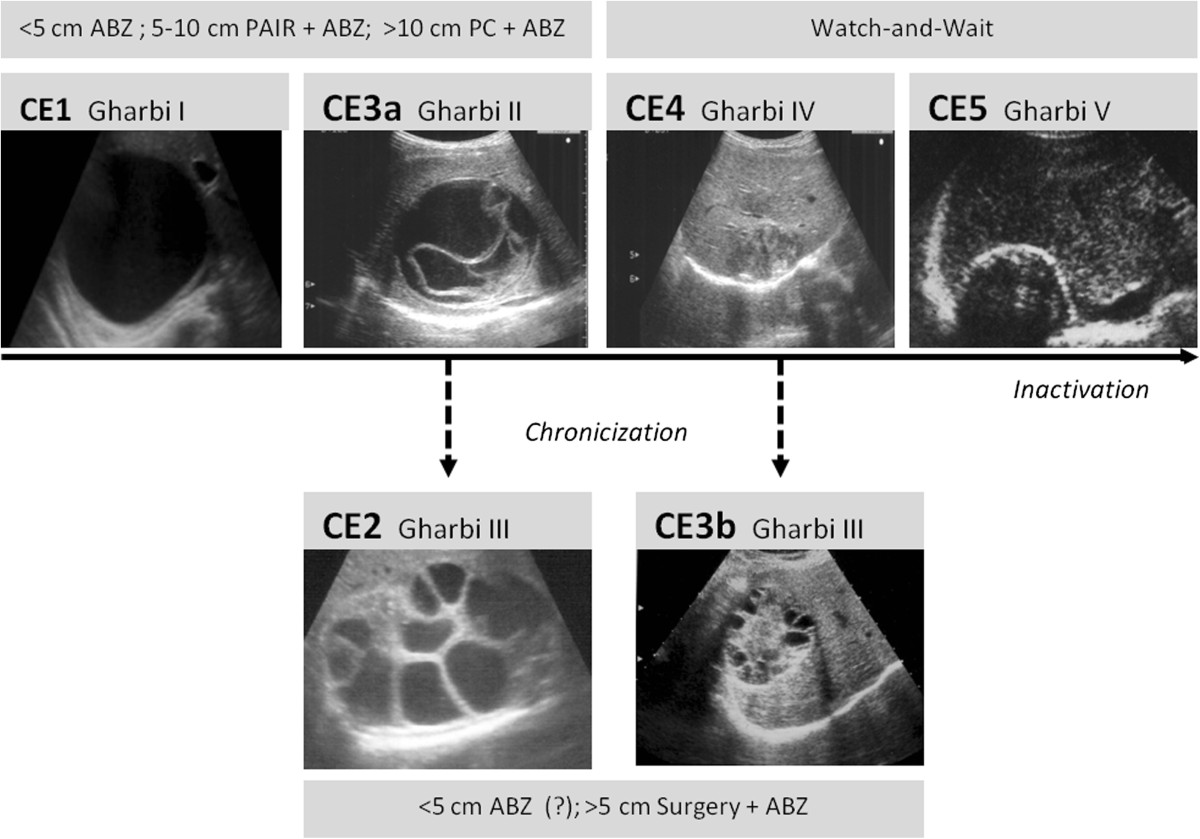
**Schematic representation of the natural history of hepatic CE and suggested treatments.** Black arrows indicate proposed cyst natural history based on clinical observation (Brunetti E., unpublished). Solid black arrows indicate natural evolution toward inactivation; black dashed arrows indicate evolution of therapy-unresponsive chronic stages. US images: cyst ultrasound classifications according to WHO-IWGE (in bold) and Gharbi [9]. As WHO-IWGE stage CE3b had not been explicitly described by Gharbi it is generally considered type III [10]. Gray boxes: suggested stage-specific approach to uncomplicated hepatic CE [4, 11, 12]. ABZ = Albendazole; PAIR = Puncture, Aspiration, Injection of scolecidal agent, Re-aspiration; PC = Permanent Catheterization.

The classification and clinical management of CE have been only recently addressed in a more harmonized manner [4]. The diagnosis of CE is based mainly on imaging methods and serology, with the latter having a complementary role [4]. Ultrasonography (US) is the mainstay of diagnosis of abdominal CE [5]. The WHO Informal Working Group on Echinococcosis (WHO-IWGE) classification, introduced in 2003, describes 5 CE stages based on US features (Figure 1): CE1 and CE2 are active cysts, CE3 are transitional, and CE4 and CE5 are inactive cysts [6]. Observations on the response to non-surgical therapy and metabolic profiles using magnetic resonance spectroscopy have shown that this classification largely reflects the biological and metabolic activity of the cysts [7, 8]. Accordingly, CE3 cysts have been further divided into CE3a, which are equally likely to be active or inactive, and CE3b, which are biologically active [7].

Cyst staging and the experience gathered in a number of referral centres allowed for a more rational allocation of uncomplicated cysts to the different available treatments (surgery, medical therapy, percutaneous interventions or “watch-and-wait” approach) (Figure 1) [4]. Treatment with albendazole (ABZ) is recommended to be administered continuously in courses of 3 to 6 months or longer, depending on cyst stage and patient characteristics [4, 13–15]. The “watch and wait” approach (WW) consists of regular US follow-up without interventions on the cyst in the absence of reactivation or complications. In our centre, this approach is currently the choice for uncomplicated inactive CE4 and CE5 cysts [4, 6, 16–18]. CE3b cysts are poorly responsive to non-surgical treatment [8, 11] and often present as indolent, asymptomatic or paucisymptomatic lesions that may not warrant surgery unless complicated. There is a lack of evidence supporting indications for treatment for these cyst stages. Often, after an attempted treatment with ABZ, which almost inevitably results in failure (i.e. relapse occurring shortly after inactivation obtained at the end of treatment) [19], in our centre CE3b cysts are allocated to the WW approach, if the patient agrees to comply with regular follow-up.

In this study we compared the behaviour of and occurrence of complications in CE3b cysts treated with ABZ with those managed with the WW approach, in a cohort of patients seen in our centre over 27 years.

## Methods

### Ethics statement

All patients gave their written informed consent to data treatment and the study was approved by the Ethics Committee of IRCCS San Matteo Hospital Foundation, Pavia, Italy.

### Data extraction and inclusion criteria

This is a retrospective, comparative study of medical treatment vs WW of patients with hepatic CE3b cysts. Clinical and demographic data of CE patients seen in our WHO Collaborating Centre for Cystic Echinococcosis from January 1985 through December 2012 were extracted from the Echinococcosis Database (FileMaker Inc., Santa Clara, CA, USA) and double-checked in the paper-based archive. Only patients with at least one hepatic CE3b cyst on first consultation and with a minimum of two follow-up visits were included in the analysis. In the case of patients diagnosed with multiple CE3b cysts, the most active cyst, i.e. the cyst with higher relapsing rate, was considered in the analysis. To reduce possible confounding factors, patients harbouring a CE3b cyst together with CE1, CE2, or CE3a cysts were excluded from the study, while those harboring a CE3b cyst together with one or more CE4 or CE5 cysts were included. Follow-up was defined as more than one consultation independent of time between visits. Data extracted included patient’s demographic data, geographical origin, cyst number, size, stage and location, time to inactivation (i.e. evolution from CE3b to inactive CE4-CE5 stage), time to relapse (i.e. reappearance of daughter vesicles in the cysts that had reached the inactive stage CE4 or CE5), treatments, and complications.

Patients who had not had a consultation during the last two years of the studied period (i.e. in 2011 and 2012) or longer, were contacted by telephone to inquire about their condition and reason for not coming to follow-up visits. Data collected by telephone were not included in the statistical analysis.

### Cysts characteristics and classification of follow-up events

Patients were evaluated by US at each follow-up visit by an Infectious Diseases clinician with long-standing experience in clinical ultrasound (EB). For records post-2003, cysts were staged according to the standardized WHO-IWGE ultrasound classification [6]. Cysts diagnosed prior to this date were classified according to Gharbi classification [9]. In these cases, Type III cysts were double checked in the photo archive and those considered as CE3b stage were included in the analysis (Figure 1). Cyst size at first consultation and before any complication event was classified as: S <5 cm, M = 5–10 cm, L >10 cm, based on the largest diameter and according to the WHO-IWGE classification [6].

**Figure 2:**
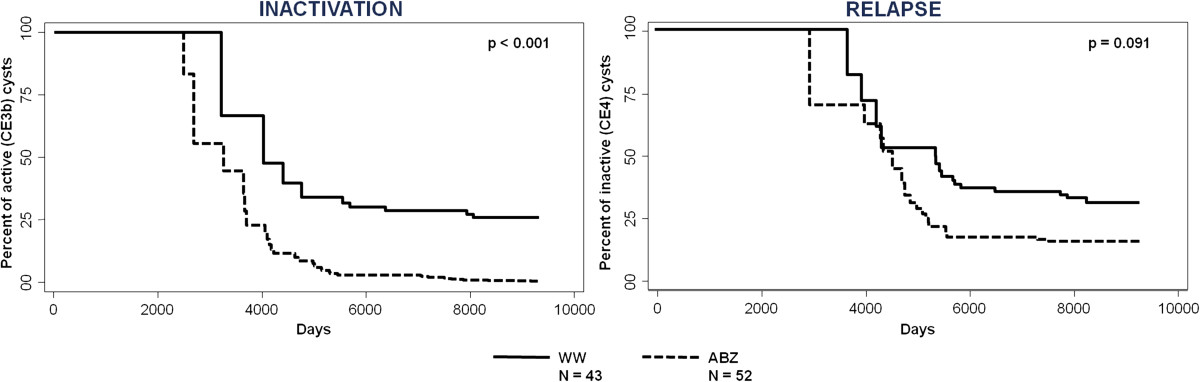
**Patients grouped by clinical management.**

The majority (58%) of CE3b cysts seen in our centre were treated with several courses of ABZ followed by at least 24 months of WW. This length of observation in the absence of treatment (≥24 months) was considered long enough to avoid confounding effects (“carry-over effect”) of the ABZ treatment on the outcome due to the natural history of the cyst during the WW period [20, 21]. Patients were therefore grouped as “ABZ only”, “ABZ/WW”, and “WW only” as schematized in Figure 2.

Complications were divided into mild, severe and lethal. Complications considered unrelated with ABZ intake were as follows. Mild: abdominal pain (experienced during periods of WW), gastrointestinal symptoms such as nausea and vomiting (experienced during periods of WW), and allergic cutaneous manifestations; severe: cyst rupture, development of biliary fistula, infection of the cyst cavity, secondary dissemination, and anaphylactic shock. Adverse events considered likely to be associated with ABZ treatment were as follows. Mild: alopecia, self-limiting, up to 5-fold increase in liver enzymes, rash, abdominal pain (experienced during ABZ intake), gastrointestinal symptoms such as nausea and vomiting (experienced during ABZ intake), and headache; severe: bone marrow suppression and liver damage [22, 23]. To our knowledge, only one case of death related to ABZ treatment has been reported in the literature [24].

### Statistical analysis

Univariate and multivariate analysis were performed to investigate the effect on outcome (inactivation or relapse) of variables such as age, sex, origin, treatment, cyst size and presence of other echinococcal hepatic cysts using a multiple failure Cox proportional hazard model. Age, treatment, cyst size and presence of other cysts were treated as time-dependent covariates. Periods of ABZ treatment and periods of WW follow-up (treatments groups, as illustrated in Figure 2) were considered separately, but taking into account that periods were clustered across subjects. For every variable, robust standard errors were calculated to correct for correlation between different treatment periods (ABZ or WW) of the same subject. Thus, a patient could act as his own control during different treatment periods (ABZ or WW). In addition, comparability between groups was also guaranteed by the use of a multivariate analysis.

Differences in cumulative time to inactivation and time to relapse between treatment groups were investigated using Kaplan-Meyer estimator. Incidence of complications were calculated together with their 95% CI and compared between treatment groups using an exact binomial test. All analyses were performed using STATA software (Stata Corporation, College Station, TX, USA). A p-value ≤0.05 was considered significant.

## Results

Data from 60 patients, seen over 27 years (1985–2012) in our centre and fulfilling the inclusion criteria, were extracted from the database. Median follow up was 43 months (IQR 10.7-141.5, range 2–306). Patients included were 34 males and 26 females, with a mean age of 43.6 years (range 8–75). Forty-six (77%) patients were from Italy, of whom 28% were from the endemic region of Sicily; the remaining 14 (23%) patients were immigrants, mainly from North Africa and Eastern Europe. The most frequent hepatic location was the VII segment(19 cysts, 31%). Seventeen (28%) patients had also hepatic inactive cysts (CE4 and CE5) in addition to the CE3b considered for the analysis. Only one patient harbored two CE3b cysts. Size at diagnosis was available for 50 CE3b cysts: S =7 (14%); M = 29 (58%); L = 14 (28%).

**Figure 3:**
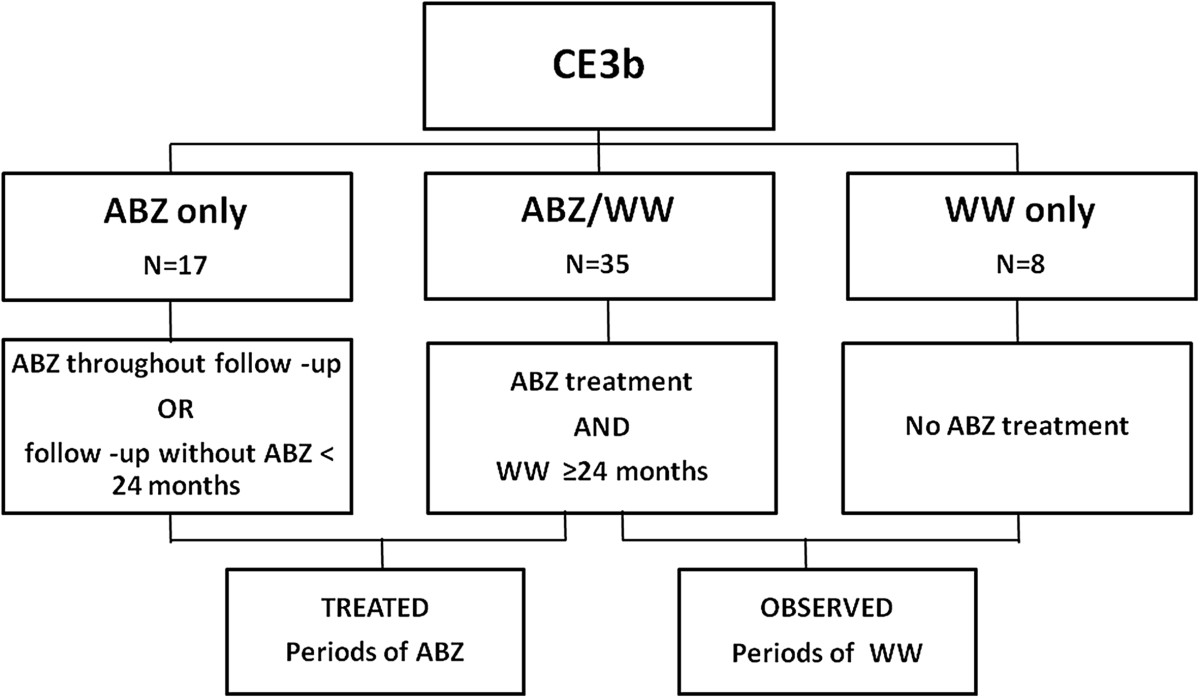
**Kaplan Meier survival curve of inactivation and relapse.**

Patients classification according to clinical management is detailed in Figure 2. Median cumulative length of ABZ intake was 12.2 months (IQR 4.2-38.3), while median WW observational period was 48.8 months (IQR 30.8-116.9). Of the 17 patients who received ABZ throughout the follow up, 7 reached stable inactivation, 1 became inactive but relapsed once and then remained CE3b, and 9 remained unchanged. In this group, one of the patients with a cyst that reached inactivation had been also treated with PAIR while receiving ABZ. Of the 8 patients who received WW only, none became spontaneously inactive. Of the 35 patients who received periods of ABZ interspaced by periods of ≥ 24 months WW, 3 reached stable inactivation, 19 became inactive but relapsed (once n = 10, twice n = 4, three times n = 4, four times n = 1) and 13 remained CE3b. In this group, of the patients who reached stable inactivation, one had also been treated with percutaneous drainage. Of the patients who relapsed, one received PAIR, one received PAIR followed by surgery, and one underwent radiofrequency thermal ablation during the observation period [25]. Of the patients who remained CE3b, one also underwent PAIR and one was surgically treated. Median time to inactivation and time to relapse are shown in Table 1.The univariate and multivariate analysis showed that ABZ treatment was positively associated with inactivation (hr 5.50, CI 2.61-11.60, p < 0.001, univariate analysis; hr 7.18, CI 2.66-19.40, p = 0.001, multivariate analysis), while Italian origin was negatively associated with inactivation (hr 0.26, CI 0.09-0.76, p = 0.01, univariate analysis; hr 0.29, CI 0.08-0.96, p = 0.04, multivariate analysis). None of the variables analyzed were associated with relapse. The influence of ABZ treatment and WW approach on inactivation and relapse are depicted in Kaplan-Meier survival plots (Figure 3). Significantly more cysts reached inactivation during ABZ intake and in a shorter time than during WW periods. Additionally, no difference was found in rate and time to relapse between cysts during ABZ or WW periods.

**Table 1:** **Evolution of cysts according to treatment approach over time**

Outcome	ABZ (n = 17)*				ABZ/WW (n = 35)*				
	N	Time to inactivation	Time to relapse	Months of ABZ	N	Time to inactivation	Time to relapse	Months of ABZ	Months of WW
Inactivation	7	4 (4–9.5)		6 (3.75-7.25)	3	8 (7.5-9.5)		6 (4–7.5)	30 (25–39)
Relapse	1	6	9	3	19	8 (5–30.5)	8 (6–13)	22 (9.5-34.5)	143 (78–192)
Unchanged	9			5 (3.5-7)	13			10 (4–20)	62 (39–107)

*Time in months is expressed as median (IQR).

Multiple complications were experienced by 6 patients, while 8 patients had a single occurrence of a complication. The most common complication was abdominal pain (6 patients, 12 episodes), followed by rupture into the biliary tree with jaundice or compression of biliary vessels (4 patients, 7 episodes). Other complications were allergic reactions (4 cases with 2 reporting rash and 2 anaphylactic shock episodes, of difficult interpretation and without available documentation), cyst rupture (2 cases), bacterial infection of the cyst (1 case), and increased liver enzymes (2 cases) and anaemia (1 case) while on ABZ.

While on ABZ, the incidence of mild events was 0.0530 (IC 0.0194–0.1154) events per year and that of severe events was 0.0176 (CI 0.0021-0.0638) events per year. While on WW, the incidence of mild events was 0.0403 (CI 0.0193-0.0741) per year and that of severe events was 0.0403 (CI 0.0193-0.0741) per year as well. No statistically significant differences were found in the incidence of adverse events between the two treatment groups.

Of the 60 patients included in the study, 24 (40%) did not have a consultation during the last two years of the studied period (i.e. in 2011 and 2012) or longer. Of these, 11 (46%) could not be reached at the telephone number provided at the time of the visit. Of the 13 patients (54%) who could provide information, 4 (31%) returned for a visit in late 2013: one patient was diagnosed with a relapse seven years after the last visit, one remained a stable CE5, and two remained CE3b three years after the last visit. Of the 9 patients who were only interviewed by telephone (69%), one underwent surgery in another hospital as he wanted the cyst removed and was not evaluated further since then, one patient died for reasons unrelated to CE, two patients declared to be followed in another hospital closer to their residence, and all other patients did not seek further medical advice because they declared to be asymptomatic and deemed further visits unnecessary.

## Discussion

To our knowledge, this is the first study on this subject in the literature, although with the limitations of a retrospective study on relatively small numbers of subjects. However, these figures are not negligible when taking into account that CE is a neglected disease with low prevalence and extremely heterogeneous clinical presentation and management practices, and our results come from a single referral centre experience. Taken together, they provide initial evidence on the feasibility of a WW approach to CE3b cysts in selected circumstances.

The introduction of the WHO-IWGE classification of echinococcal cysts, followed by the expert consensus for CE diagnosis and treatment, has provided a long needed framework for the clinical management of this condition [4, 6]. Nevertheless, the current management of CE is still largely based on expert opinion and moderate to poor evidence [4]. Four treatment modalities are currently available for CE (surgery, percutaneous treatments, medical therapy with benzimidazole derivatives such as albendazole, and a watch and wait approach). However, longitudinal controlled studies comparing the efficacy and effectiveness of the different treatment options for specific clinical stage are lacking. As a consequence, the issue of “best” treatment for echinococcal cysts of the liver is still controversial [4].

CE3b cysts are the least responsive to non-surgical treatments and relapse almost invariably occurs soon after ABZ discontinuation [11, 19]. However, in our experience they often show an indolent behavior with infrequent development of complications and as such they are often assigned to a “watch-and-wait” approach when a surgical intervention does not appear to be necessary and the patient agrees to comply with a regular follow-up. We analyzed treatment outcome and incidence of complications in patients with hepatic CE3b cysts managed with either albendazole or watch-and-wait approach to evaluate their effectiveness and safety.

Our results show that during ABZ treatment cysts showed a higher probability to become inactive and inactivation occurred in a shorter time period compared to periods during which cysts were managed expectantly. In addition, the probability of and time to relapse after initial ABZ-induced inactivation did not significantly differ during ABZ or WW periods. This is unlikely to be related to any percutaneous interventions also performed in some patients, as the final outcome of the 5 patients who received this treatment was indeed stable inactivation in only 1 case. These results indicate that treatment with ABZ does not induce permanent inactivation of CE3b cysts, at least not in our cohort.

While temporary response to ABZ treatment was predictable, the correlation between outcome and geographical origin of patients is difficult to explain. This different behaviour is likely not due to the presence of different CE genetic strains in different areas as recent investigations have shown that the G1-G3 *E. granulosus sensu stricto* genotype complex is largely prevalent in all endemic areas [26–31]. Cyst size was unrelated to treatment outcome, in contrast with what reported by Stojkovic et al. [32].

We found no statistically significant difference in the rate of complications during ABZ or WW. Thus, the decision not to treat uncomplicated CE3b cysts does not seem to have an increased risk of a mild or severe adverse event in our series, at least over a median observation period without treatment of 48.8 months. One of the two patients who reported an (undocumented) anaphylactic reaction during WW was receiving amoxicillin-clavulanate at that time, a drug that might have caused this event [33]. As for the second patient reporting an episode of shock, this occurred before she presented to our clinic. Because we never witnessed the event, the actual occurrence of a true anaphylactic shock should be taken cautiously.

Surgery was performed on 3 patients during WW period, due to rupture into the biliary tree (n = 2) and cyst infection (n = 1). However, we observed rupture into the biliary tree with jaundice also during ABZ intake periods, although this did not result in the decision to refer the patient to surgery due to patient-related issues.

Taken together, our results suggest that a WW approach to CE3b cysts is feasible. Nevertheless, one should be cautious in drawing conclusions on the applicability of WW for all patients with CE3b cysts. The loss of CE patients to follow-up is a problem commonly faced by clinicians. In our series, of those patients who did not have a consultation during the last two years of the studied period (i.e. in 2011 and 2012) or longer and could be reached by telephone, 38% declared that they did not seek further medical advice because they were asymptomatic and they did not consider a control visit necessary. However, it is extremely important to stress that a constant, long-term follow-up of patients with CE3b cysts is mandatory when surgery is not considered the first choice based on a patient-tailored approach, and clearly, a good doctor-patient relationship and a better explanation of the necessity of regular follow-up of even asymptomatic CE cysts should be offered. Indeed, major complications such as rupture into the biliary tree may develop during both WW and ABZ treatment periods, therefore non-surgical approaches, including WW, should be considered only for those patients who adhere strictly to the follow-up schedule.

## Conclusions

In conclusion, our study shows that ABZ treatment does induce inactivation of CE3b cysts, but this is only temporary. Additionally, during WW, CE3b cysts remain stable over time. As the rate of adverse events during ABZ treatment and WW observation did not differ significantly, expectant management might represent a viable option for patients with asymptomatic CE3b cysts that do not warrant surgery and who can comply with regular follow-up. In the majority of cases, patients with CE3b cysts have been receiving treatment(s) with ABZ interrupted by observation periods without treatment. Therefore, a direct comparison between those only treated with ABZ and those only untreated was not possible. Longer prospective observation of a larger cohort after allocation to clearly distinct management options is needed to confirm our findings.

## Authors’ original submitted files for images

Below are the links to the authors’ original submitted files for images. Authors’ original file for figure 1 Authors’ original file for figure 2 Authors’ original file for figure 3

## Abbreviations

ABZ: Albendazole
CE: Cystic cchinococcosis
PAIR: Puncture, aspiration, injection, re-aspiration
WHO-IWGE: World Health Organization Informal Working Group on Echinococcosis
WW: Watch and wait.
