# Supplementary figures and images for: Medical treatment versus “Watch and Wait” in the clinical management of CE3b echinococcal cysts of the liver
Source: BMC Infect Dis. 2014 Sep 9;14:492. doi: 10.1186/1471-2334-14-492 (PMC4164709; doi:10.1186/1471-2334-14-492)

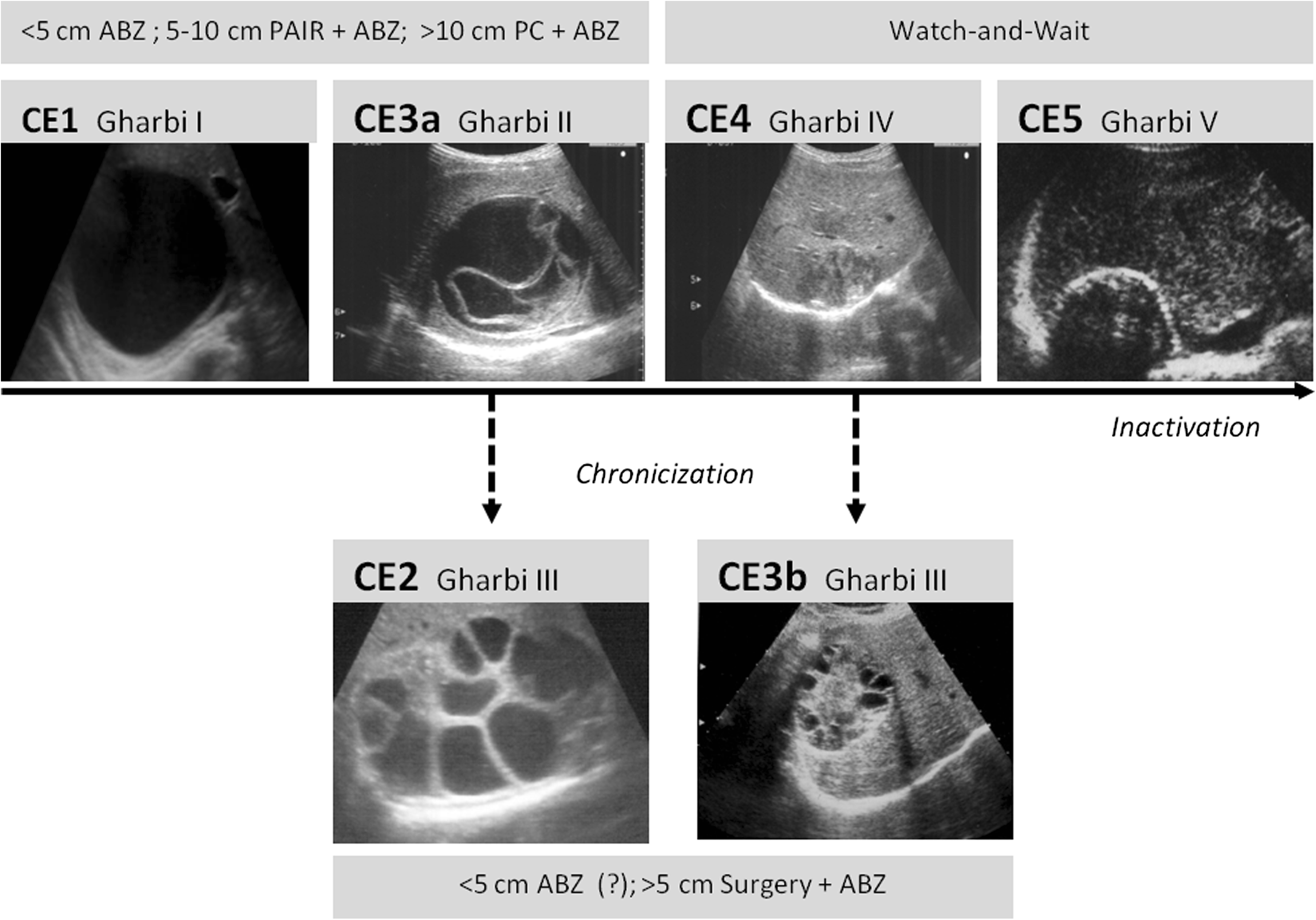

Supplement: Supplementary file 1 — Authors’ original file for figure 1 [file 12879_2014_3792_MOESM1_ESM.tif]

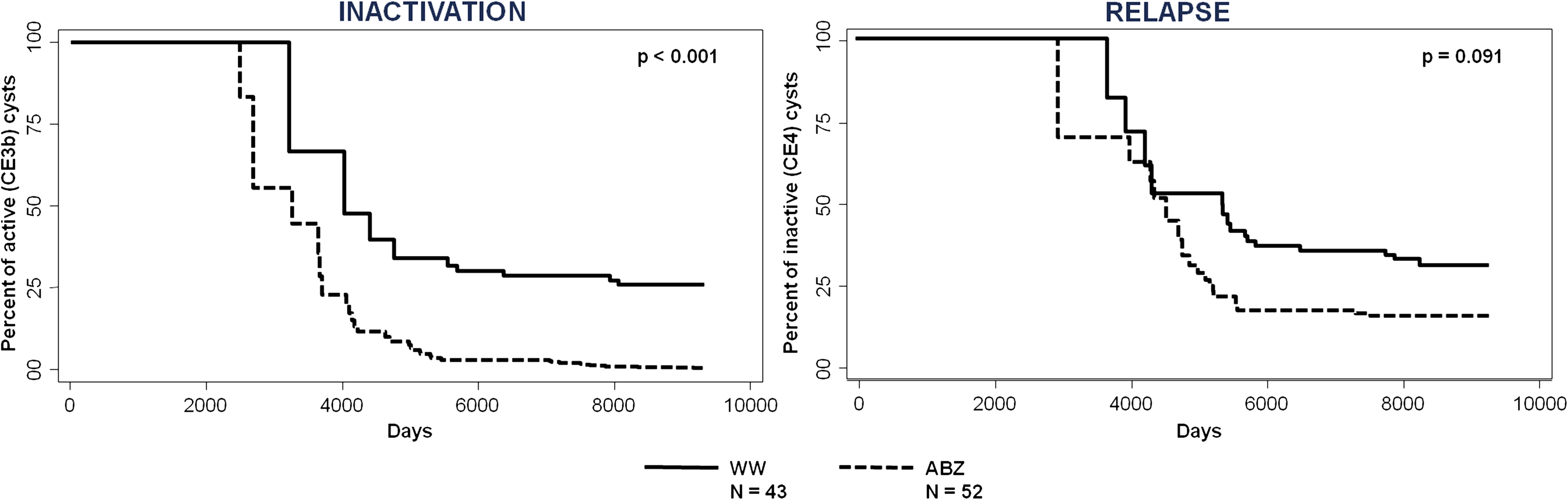

Supplement: Supplementary file 2 — Authors’ original file for figure 2 [file 12879_2014_3792_MOESM2_ESM.tif]

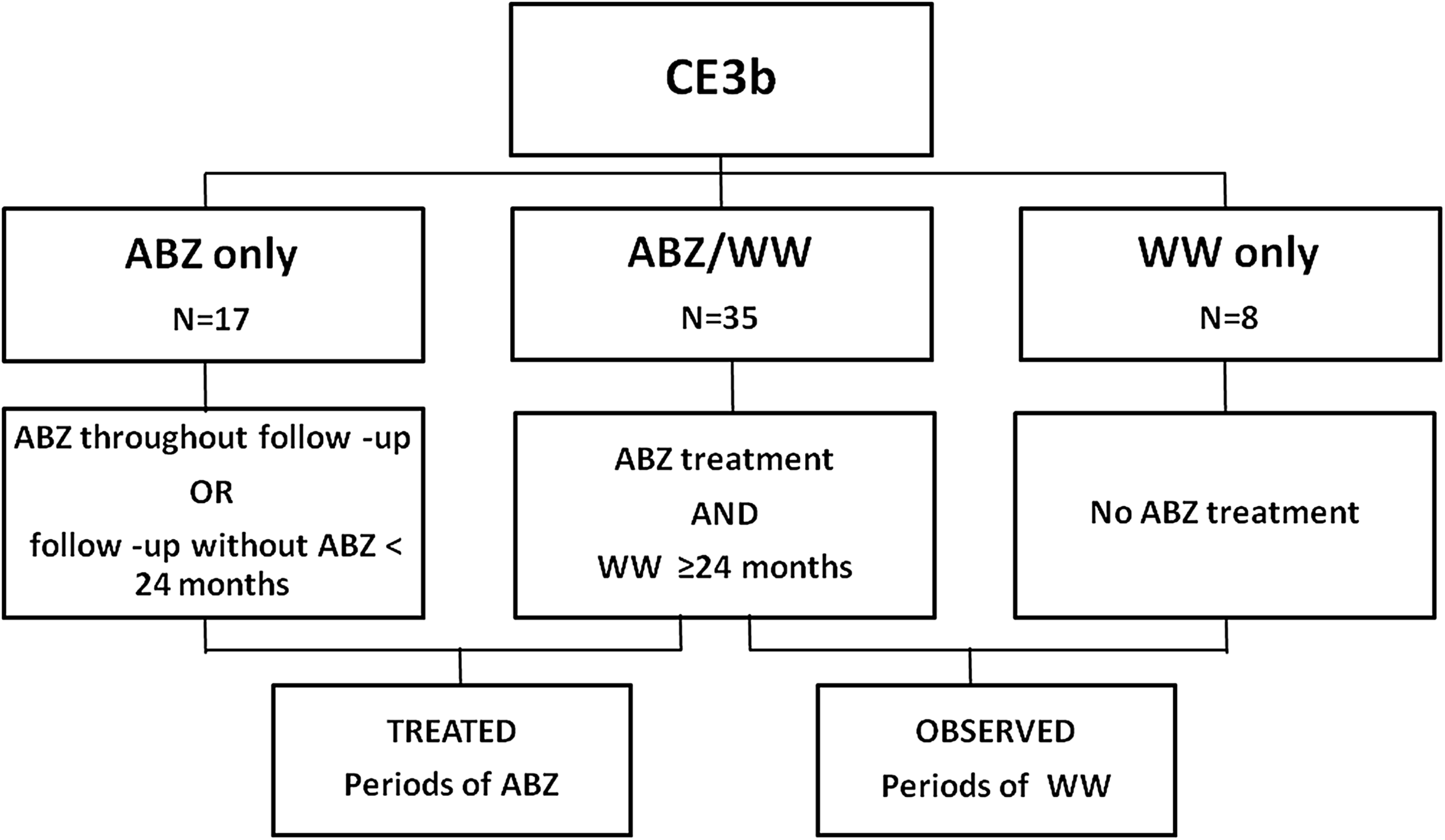

Supplement: Supplementary file 3 — Authors’ original file for figure 3 [file 12879_2014_3792_MOESM3_ESM.tif]
